# Enhanced production of pinosylvin stilbene with aging of *Pinus strobus* callus and nematicidal activity of callus extracts against pinewood nematodes

**DOI:** 10.1038/s41598-022-04843-6

**Published:** 2022-01-14

**Authors:** Hyo Bin Koo, Hwan-Su Hwang, Jung Yeon Han, Eun Ju Cheong, Yong-Soo Kwon, Yong Eui Choi

**Affiliations:** 1 Department of Forest Resources, College of Forest and Environmental Sciences, Kangwon National University, Chuncheon, 200-701 Republic of Korea; 2 College of Pharmacy, Kangwon National University, Chunchon, 200-701 Republic of Korea

**Keywords:** Biotechnology, Plant sciences

## Abstract

Pinosylvin stilbenes are phenolic compounds mainly occurring in the Pinaceae family. We previously reported that the accumulation of two pinosylvin stilbene compounds, dihydropinosylvin methyl ether (DPME) and pinosylvin monomethyl ether (PME), in *Pinus strobus* trees was highly enhanced by infection with pine wood nematodes (PWNs: *Bursaphelenchus xylophilus*), and these two compounds showed strong nematicidal activity against PWNs. In this work, we established a system of pinosylvin stilbene (DPME and PME) production via the in vitro culture of *P. strobus* calli, and we examined the nematicidal activity of callus extracts. Calli were induced from the culture of mature zygotic embryos of *P. strobus*. Optimized growth of calli was obtained in 1/2 Litvay medium with 1.0 mg/L 2,4-D and 0.5 mg/L BA. DPME and PME accumulation did not occur in nonaged (one-month-old) calli but increased greatly with prolonged callus culture. The concentrations of DPME and PME in three-month-old dark-brown calli were 6.4 mg/g DW and 0.28 mg/g DW, respectively. The effect of methyl jasmonate treatment on the accumulation of DPME and PME was evaluated in cell suspension culture of *P. strobus*. However, the treatment appeared to show slight increase of DPME accumulation compared to callus browning. A test solution prepared from crude ethanol extracts from aged calli (three months old) containing 120 µg/ml DPME and 5.16 µg/ml PME treated with PWNs resulted in 100% immobilization of the adult PWNs and 66.7% immobilization of the juvenile PWNs within 24 h. However, nonaged callus extracts did not show any nematicidal activity against juvenile PWNs and showed less than 20% nematicidal activity against adult PWNs. These results indicate that pinosylvin stilbenes can be effectively produced by prolonged culture of *P. strobus* calli, can be isolated using simple ethanolic extraction, and are applicable as beneficial eco-friendly compounds with nematicidal activity against PWNs.

## Introduction

Pine wilt disease (PWD) caused by pine wood nematodes (PWNs; *Bursaphelenchus xylophilus*) is the most severe disease of forests worldwide^1^. PWN infection of pine trees occurs by migration to host trees when the pine sawyer beetle (*Monochamus* spp.) feeds on the young shoots of pine trees^2^. PWD symptoms begin with needle discoloration and eventually lead to the death of pine trees in PWN-susceptible pine species^3^. Controlling the spread of PWN and pine destruction are major economic, ecological, and environmental concerns worldwide^4^.

The biological control of PWNs has remained a challenging task for a long time because PWD is the result of a complex interaction among a nematode, a host tree and an insect vector. The current methods of controlling PWD mainly rely on chemical treatments, including nematicide implantation in the trunks of trees. The two most widely used nematicides are abamectin and emamectin benzoate, which are members of the macrocyclic lactone family^5^. Although these chemicals are highly effective against various species of nematodes, they are highly toxic to mammals and insects, which can spoil the forest environment and can cause adverse effects on human health. Moreover, the recurrent use of these nematicides induces resistant nematodes, such as *Trichostrongylus colubriformis* and *Ostertagia circumcincta,* against abamectin^6^. Hence, the invention of new nematicides should be performed continuously to identify alternative chemicals to combat PWNs. Several papers have been published on nematode control agents using plant extracts, essential oils, and volatile compounds^7,8^.

Stilbenoid phytoalexins are phenolic compounds and mainly occur in the Pinaceae family. In pine trees, pinosylvin-type stilbenes prevent wood tissues from decaying by fungi and are particularly rich in heartwood extracts^9^. There are many reports on the biosynthesis of pinosylvin stilbenes in the sapwood and needles of pine plants by abiotic and biotic treatment, such as UV‐C^10^, wounding^11^, ozone^12^, and fungal pathogens^13^. Pinosylvin-type stilbenes showed strong antibacterial, antifungal, and antifeedant activities^14–17^. Suga et al.^18^ suggested that the resistance of *Pinus* species to PWNs is attributed to the presence of these endogenous defense substances, among which pinosylvin-type stilbenes extracted either from the heartwood of *P. strobus* or the heartwood and bark of *P. palustris* have strong nematicidal activity. Recently, we reported that the accumulation of dihydropinosylvin monomethylether (DPME) and pinosylvin monomethylether (PME) is highly responsive to PWN infection and plays an important role in the PWN resistance of *P. strobus* trees^19^. The nematicidal activity of DPME and PME resulted in a developmental stage-dependent manner: PME was more toxic to adult PWNs than juveniles, whereas DPME was found to be more toxic to juvenile PWNs than adults^19^. These reports indicate that pinosylvin-type stilbenes may be good candidates for nematicidal compounds for controlling the PWD.

Plant cell culture systems are promising technologies because they are reliable alternative methods of producing high-value secondary metabolites of industrial importance and have been extensively investigated for the production of secondary metabolites^20,21^. Resveratrol and its derivatives are well-known stilbenoid compounds and have emerged as promising molecules because of their benefits to human health. Many reports have been published on the biotechnological production of resveratrol and its derivatives through cell suspension culture, particularly using grapevine cell cultures^22–25^. However, only one article reported the production of pinosylvin-type stilbene using a cell suspension culture of *Pinus sylvestris*^26^.

To optimize production, elicitation has been shown to be the most efficient way to promote the synthesis and accumulation of compounds of interest^27,28^. Methyl jasmonate (MeJA) has been reported as an important elicitor to enhance the production of various secondary metabolites in plants^29,30^. Elicitation using MeJA is also effective for the enhancement of stilbene production (resveratol) in grapevine cell culture^22–25^. There is no report on the production of pinosylvin stilbenes by MeJA treatment in pine calli and cell suspension cultures. An elicitor preparation from the pine needle pathogen *Lophodermium seditiosum* in *Pinus sylvestris* cell suspension cultures resulted in strong accumulation of pinosylvin stilbenes^26^.

Browning of callus is frequently observed in tissue cultures of woody plants, which is caused by the accumulation and oxidation of phenolic compounds^31,32^. The browning process is associated with cell disorganization and eventual cell death^33^. Pinosylvin stilbenes are typical phenolic compounds mainly found in the *Pinus* species. It is questioned whether the production of pinosylvin stilbene is associated with the browning process of callus. However, no report has been published on the production of pinosylvin stilbenes during the browning process in plant tissue culture.

Here, we established in vitro culture conditions for the induction and proliferation of *P. strobus* calli from zygotic embryo cultures and investigated the enhanced accumulation of pinosylvin stilbenes by callus aging with prolonged culture. We analyzed the effect of MeJA treatment on the production of pinosylvin stilbenes from callus. We found that ethanolic extracts of aged *P. strobus* calli after prolonged culture had strong nematicidal activity against PWNs.

## Materials and methods

### Callus induction from the culture of mature zygotic embryos of *P. strobus*

Seeds of *P. strobus* were purchased from Danong Inc. (Gyeonggi-do, Korea). All the experiments were performed in accordance with relevant institutional, national, and international guidelines and regulations. After dehusking shells from seeds, inner nuts were soaked in a 70% solution of EtOH and then transferred into 1% NaClO for 10 min and rinsed with sterilized distilled water. Then, mature zygotic embryos were dissected from the megagametophytes and cultured in vitro on semisolid callus induction medium. Approximately 15 zygotic embryos were plated onto a Petri dish cotaining 1/2 LV medium^34^ supplemented with 1.0 mg/L 2,4-dichlorophenoxyacetic acid (2,4-D). All media were also supplemented with 20 g/L sucrose and 2.8 g/L Phytagel (Sigma). The pH was adjusted to 5.7 before autoclaving at 121 °C. All the cultures were incubated at 25 °C in dark condition.

### Culture conditions of optimal proliferation of callus

Friable calli were obtained from cultured zygotic embryos after regular subculture of calli at 3-week intervals. To investigate the optimal production of calli, various culture conditions were tested. Approximately 300 mg of callus was cultured on 1/2 LV medium with various concentrations of 2,4-D (zero, 0.5, 1.0, 2.0, and 4.0 mg/L) or 1.0 mg/L 2,4-D with 0.5 mg/L BA. To investigate the effects of cytokinin types, calli were cultured onto 1/2 LV medium with various types of cytokinins (0.5 mg/L BA, zeatin, kinetin, TDZ, and 2-ip) and 1.0 mg/L 2,4-D. To investigate the effect of BA concentration, calli were cultured onto 1/2 LV medium with various concentrations of BA (0.25. 0.5, 1.0, and 2.0 mg/L) and 1.0 mg/L 2,4-D. After 4 weeks of culture, fresh and dry weights were taken. All media were also supplemented with 20 g/L sucrose and 2.8 g/L Phytagel (Sigma). The pH was adjusted to 5.7 before autoclaving at 121 °C. All the cultures were incubated at 25 °C in dark. For statistical analysis, 300 mg of callus (separated to about 12 to 15 pieces) were transferred to a Petri dish. Each treatment was conducted with three to five replicates. The experiment was repeated three times.

### MeJA treatment to callus

To investigate the effect of MeJA on the growth of calli and the production of PME and DPME, calli were transferred onto 1/2 LV medium with 1.0 mg/L 2,4-D with 0.5 mg/L BA and various concentrations (zero, 10, 50, 100 µM) of MeJA. Callus growth and DPME and PME accumulation in callus were analysed after 2 weeks of MeJA treatment. For analysis of gene expression involved in the biosynthesis of PME and DPME by MeJA treatment, calli were sampled after 2 days of MeJA treatment. To investigate the effect of MeJA on the production of PME and DPME, 500 mg of calli were transferred into 200 mL Erlenmeyer flasks containing 50 mL 1/2 LV liquid medium with 1.0 mg/L 2,4-D with 0.5 mg/L BA and with and without 100 µM MeJA. Culture flasks were agitated at 120 rpm under dark conditions. Each treatment was conducted with three replicates and the experiment was repeated three times. Cells were harvested after zero, 1, 3, and 7 days of culture, and the content of PME and DPME was analyzed by GC/MS.

### RT-PCR and qRT-PCR analysis in MeJA treated callus

Total RNA was isolated from calli, treated with or without MeJA treatment by the RNeasy Plant Mini Kit for RT-PCR (Qiagen, Germany), and converted to cDNA using M-MLV reverse transcriptase (Invitrogen, USA). RT-PCR was conducted using first-strand cDNA as a template with the following conditions: 95 °C for 3 min; 30 cycles at 95 °C for 30 s, 55 °C for 30 s, and 72 °C for 2 min; and a final extension at 72 °C for 10 min. The primers used for RT-PCR analysis used in this study are listed in Table S1. RT-PCR analysis was repeated three times, and representative data are shown in Fig. 8.

Rotor-Gene Q with QuantiTect SYBR Green PCR Kit (Qiagen, Germany) was used for qPCR analysis. Real-time cycler conditions were 95 °C for 15 min, followed by 40 cycles of 94 °C for 15 s, 60 °C for 30 s, and 72 °C for 30 s. qPCR analysis was performed with at least three replicates, and the data are presented as the average relative quantities ± SEs. The relative expression value of each gene was calculated using the 2^-ΔΔ^CT method^35^. The β-actin gene of *P. strobus* was used for normalization. The primers for qPCR analysis used in this study are listed in Table S1.

### GC–MS analysis of DPME and PME in callus

Calli were subcultured onto 1/2 LV medium with 20 g/L sucrose, 1.0 mg/L 2,4-D, and 0.5 mg/L BA at 2-week intervals. Calli were not subcultured until 3 months to induce callus aging. Calli was sampled after one, two, and three months of culture. To measure the contents of PME and DPME compounds by gas chromatography-mass spectrometry (GC–MS), calli were air-dried at 50 °C in a drying oven. Milled powder (200 mg) from each of the samples was soaked in 100% methanol (1 ml) and sonicated for 30 min at a constant frequency of 20 kHz at 25 °C. The supernatant obtained by centrifugation (15,000 × *g* for 10 min) was subsequently filtered using a SepPak C-18 Cartridge (Waters) to remove debris. The filtered aliquots were analyzed by GC (Agilent 7890A) linked to an inert MSD system (Agilent 5975C) with its Triple-Axis detector and equipped with an HP-5MS capillary column (30 m × 0.25 mm, film thickness 0.25 mm). The injection temperature was 250 °C, and the column temperature program was as follows: 70 °C for 4 min, followed by an increase to 220 °C at a rate of 5 °C min^−1^, heating at 4 °C min^−1^ up to 320 °C, and a hold at 320 °C for 5 min. The carrier gas was He, and the flow rate was 1.2 ml min^−1^. The interface temperature was 300 °C, with a split/splitless injection (10:1). The temperature of the ionization chamber was 250 °C, and ionization was performed by electron impact at 70 eV. The standards of PME and DPME used in GC–MS analysis were purchased from Sigma–Aldrich Co. Each analysis was conducted with three replicates, and the experiment was repeated three times.

### Nematicidal activity of callus extracts

PWNs were cultured on potato dextrose agar medium (PDA) with *Botrytis cinerea* for 2 weeks in darkness. The proliferated PWNs were separated using the Baerman funnel method^36^. To estimate the nematicidal activity of callus extracts, aged calli with dark brown color and nonaged calli with faded yellow color were dried in an oven at 50 °C for 24 h. The dried calli were ground well using a mechanical blender into fine powder. The powdered callus (4 g) was dipped in a 50 mL conical tube containing 100% EtOH and sonicated for 30 min at a constant frequency of 20 kHz at 25 °C. After centrifugation of the tube (5000 × *g* for 10 min), the supernatant was collected, and then EtOH was evaporated to obtain the concentrated extracts. The callus extracts were dissolved in water containing 10 mg/mL 2-hydroxypropyl-β-cyclodextrin (HP-β-CD), which was used as an emulsifier to dissolve hydrophobic stilbene by He et al.^36^. The final tested concentration of DPME using EtOH extracts of three-month-old calli with dark brown color was adjusted to 120 μg/mL by gradual dilution with HP-β-CD after GC analysis. The same dilution of EtOH extracts of one-month-old calli with a faded yellow color was achieved with HP-β-CD solution. PWNs at the adult and juvenile stages obtained using the same protocol presented by Hwang et al. ^19^ were inoculated in each treatment solution in 96-well plates and cultured for 24 h at 25 °C. About 50 adults and 80 J2 juvenile stage nematodes were inoculated in each test solution. HP-β-CD solution without PME and DPME was used as control. HP-β-CD (10 mg/mL) solution without callus extracts was used as a control. The immobilization of PWNs was counted by a light microscope. The experiment was performed in triplicates and repeated five times.

### Statistical analysis

Statistical analysis was performed using Microsoft Excel and SPSS software. The significance of callus production was tested by analysis of variance (ANOVA). Significant differences among means were identified using Tukey’s test at a significance level of 5%.

## Results

### Induction and proliferation of calli from the culture of mature zygotic embryos of *P. strobus*

**Figure 1:**
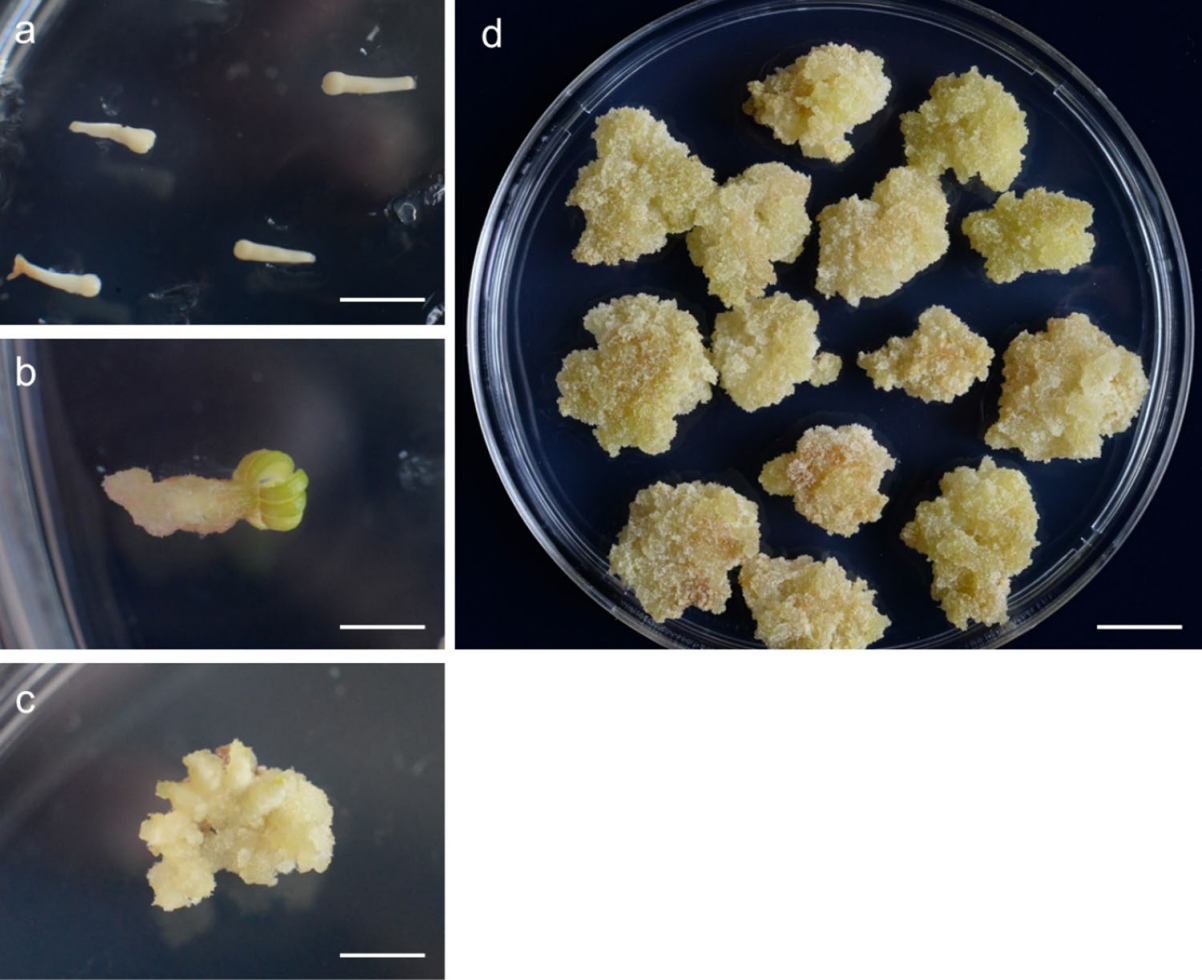
Induction and proliferation of calli from mature zygotic embryo culture on 1/2 LV medium with 1 mg/L 2,4-D and 0.5 mg/L BA. (**a**) Culture of mature zygotic embryos of *P. strobus.* (**b**). Induction of calli on the surfaces of zygotic embryos after two weeks of culture. (**c**) Callus induction after subculture on new 1/2 LV medium with 1 mg/L 2,4-D and 0.5 mg/L BA. (**d**) Actively proliferated calli on 1/2 LV medium with 1 mg/L 2,4-D and 0.5 mg/L BA after 4 weeks of culture. Bar in (**a)** = 4 mm. Bar in (**b**) = 2 mm. Bar in (**c**) = 2 mm. Bar in (**a**) = 15 mm.

Mature zygotic embryos (Fig. 1a) were cultured on 1/2 LV medium with 1.0 mg/L 2,4-D. Calli were produced on the surfaces of zygotic embryos after 3 weeks of culture (Fig. 1b). The enlarged zygotic embryos were subcultured onto the same medium and turned to callus mass after three weeks of culture (Fig. 1c). Highly friable callus masses were obtained after consecutive subculture on the same medium with 1.0 mg/L 2,4-D five times at two-week intervals (Fig. 1d).

**Figure 2:**
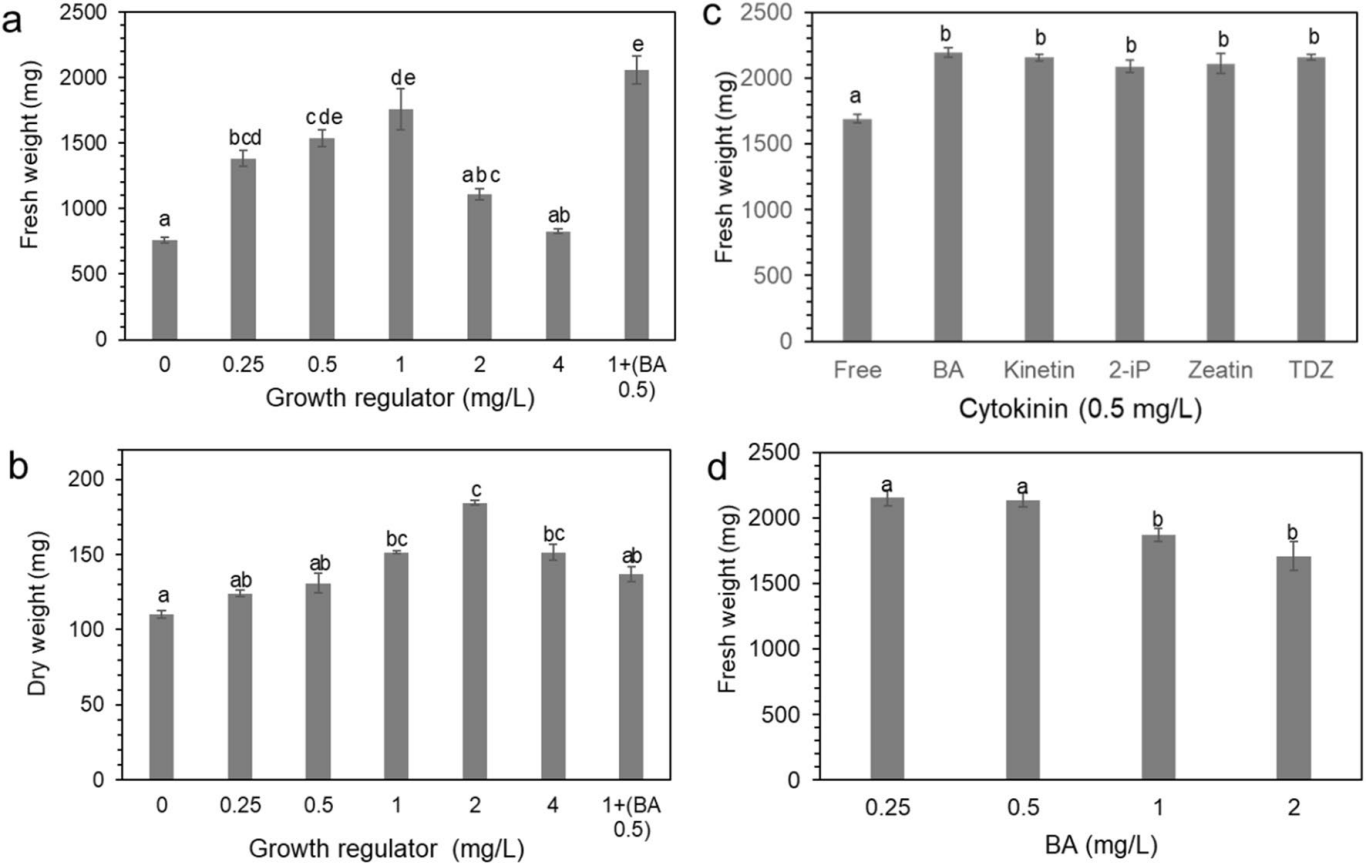
Fresh and dry weights of *P. strobus* calli proliferated under various culture conditions. (**a**) Fresh weight of calli cultured on various concentrations of 2,4-D (zero, 0.25, 0.5, 1.0, 2.0, 4.0 mg/L) and 1.0 mg/L 2,4-D with 0.5 mg/L BA after 4 weeks of culture. (**b**) Dry weight of calli cultured on various concentrations of 2,4-D (zero, 0.25, 0.5, 1.0, 2.0, 4.0 mg/L) and 1.0 mg/L 2,4-D with 0.5 mg/L BA after 4 weeks of culture. (**c**) Fresh weight of calli cultured on 1/2 LV medium containing 1.0 mg/L 2,4-D with different types of cytokinins (BA, TDZ, zeatin, kinetin, and 2-iP, 0.5 mg/L) after 4 weeks of culture. Means with the same letter are not significantly different from each other (P > 0.05 one-way ANOVA). Error lines represent ± SE of the mean of three independent experiments, each performed in technical triplicates.

Calli were transferred onto various concentrations (0, 0.25, 0.5, 1.0, 2.0, and 4.0 mg/L) of 2,4-D to evaluate the optimal concentration of 2,4-D. After 3 weeks of culture, fresh weight and dry weight were calculated (Fig. 2a,b). Calli actively proliferated at 0.5 and 1.0 mg/L 2,4-D (Fig. 2a,b). Interestingly, combined treatment with BA and 2,4-D stimulated the growth of calli (fresh weight) compared to 2,4-D alone. However, there were no differences in the growth of calli induced by the different types of cytokinins (0.5 mg/L BA, zeatin, kinetin, TDZ, and 2-ip) with 1.0 mg/L 2,4-D (Fig. 2c). When calli were cultured on medium with 1.0 mg/L 2,4-D and different concentrations of BA, 0.25 and 0.5 mg/L BA was better than 1.0 and 2.0 mg/L BA for callus production (Fig. 2d).

### Enhanced DPME and PME accumulation by prolonged culture of calli

**Figure 3:**
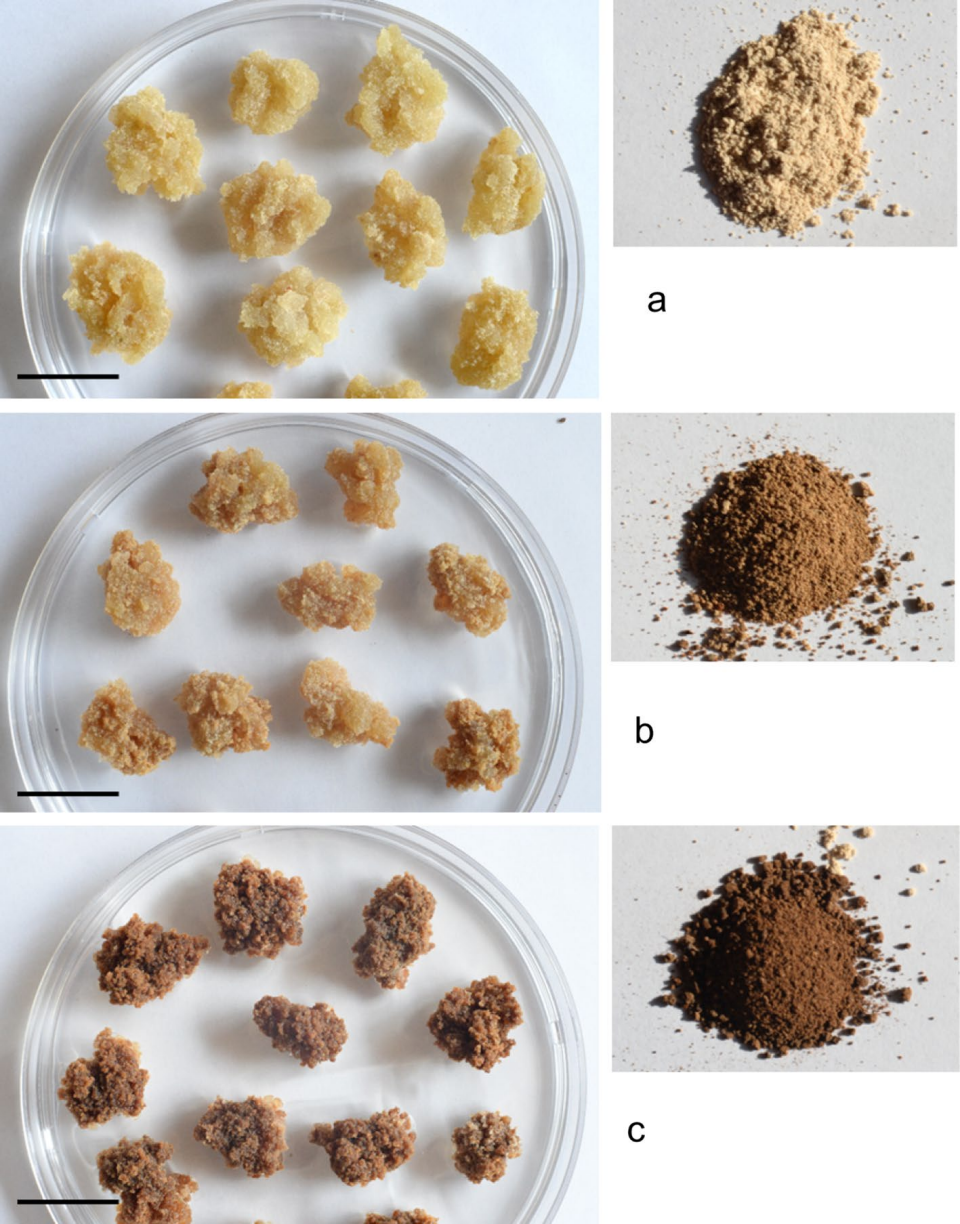
Photos of callus its ground powders after drying. (**a**) Faded yellow callus after one month of culture on 1/2 LV medium with 1.0 mg/L 2,4-D with 0.5 mg/L BA. (**b**) Brown callus after 8 weeks of culture on 1/2 LV medium with 1.0 mg/L 2,4-D with 0.5 mg/L BA. (**c**) Dark brown callus after 12 weeks of culture on 1/2 LV medium with 1.0 mg/L 2,4-D with 0.5 mg/L BA. Photos on the right sides (**a–c**) are ground powders after drying. Bars in (**a–c**) = 20 mm.

**Figure 4:**
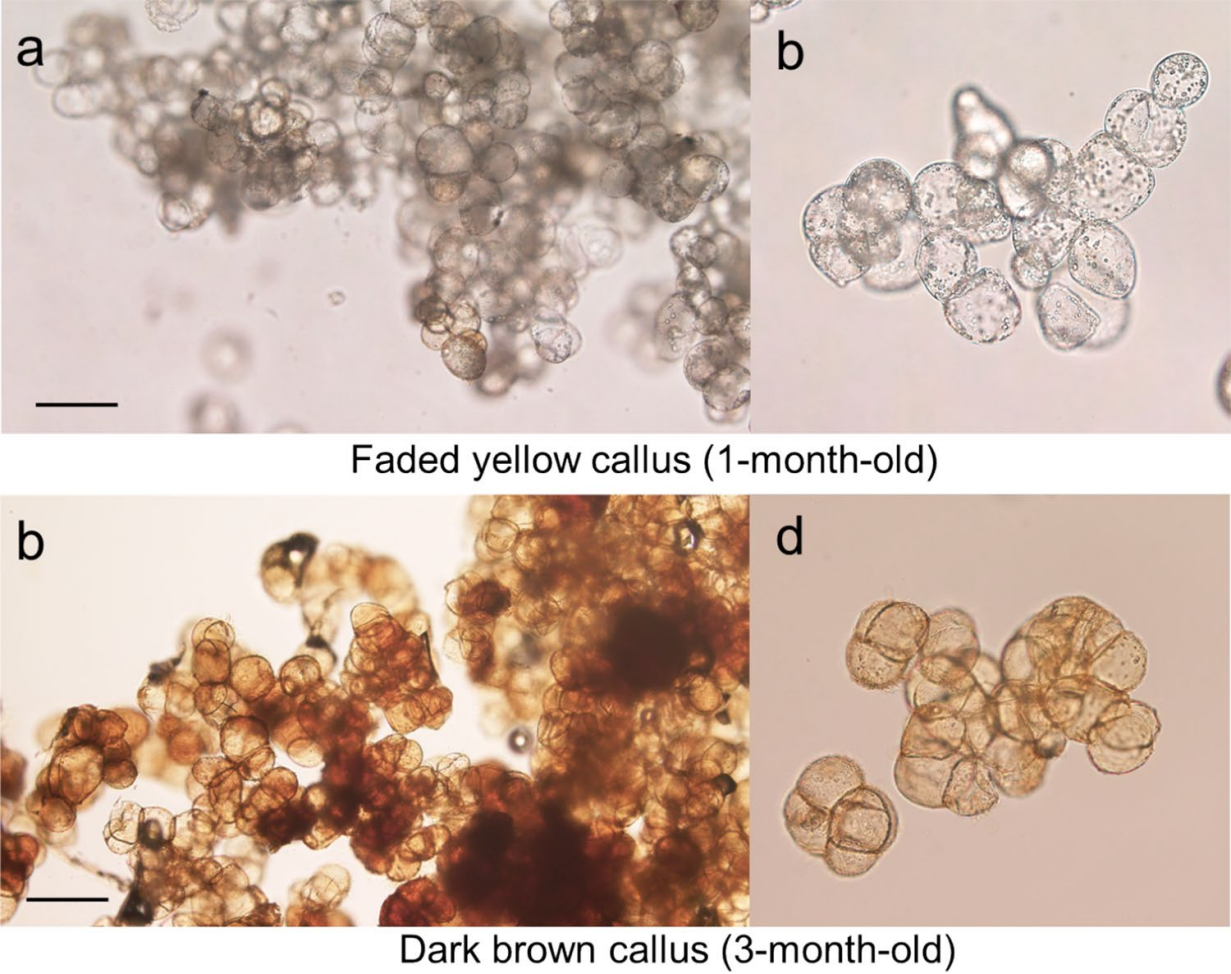
Light microscopic observation of yellow and dark brown calli. (**a**) Faded yellow callus after one month of culture constituted with cell mass with spherical cell structure with dense cytoplasm. (**b**) Callus constituted with cell mass with spherical cell structure with brown pigment. Right side of photos are enlarged views. Bars in (**a,b**)  = 50 μm.

For proliferation of calli, *P. strobus* calli were subcultured onto 1/2 LV medium with 1.0 mg/L 2,4-D and 0.5 mg/L BA at 3-week intervals. When the *P. strobus* callus was not subcultured until 3 months, the color of the callus turned pale yellow after one month of culture (Fig. 3a), turned brown after 2 months of culture (Fig. 3b), and turned dark brown after 3 months of culture (Fig. 3c). Calli with different colors were sampled and milled after drying. The color of the dried callus powders still had the same color as undried fresh callus masses (Fig. 3a–c). Microscopic observation of the callus revealed that a 1-month-old faded yellow callus was composed of aggregated cells with a spherical structure with dense cytoplasm (Fig. 4a,b). However, three-month-old brown calli were composed of cells with a brown pigment near the cell walls and less dense cytoplasm than the cells of faded yellow calli (Fig. 4c,d).

**Figure 5:**
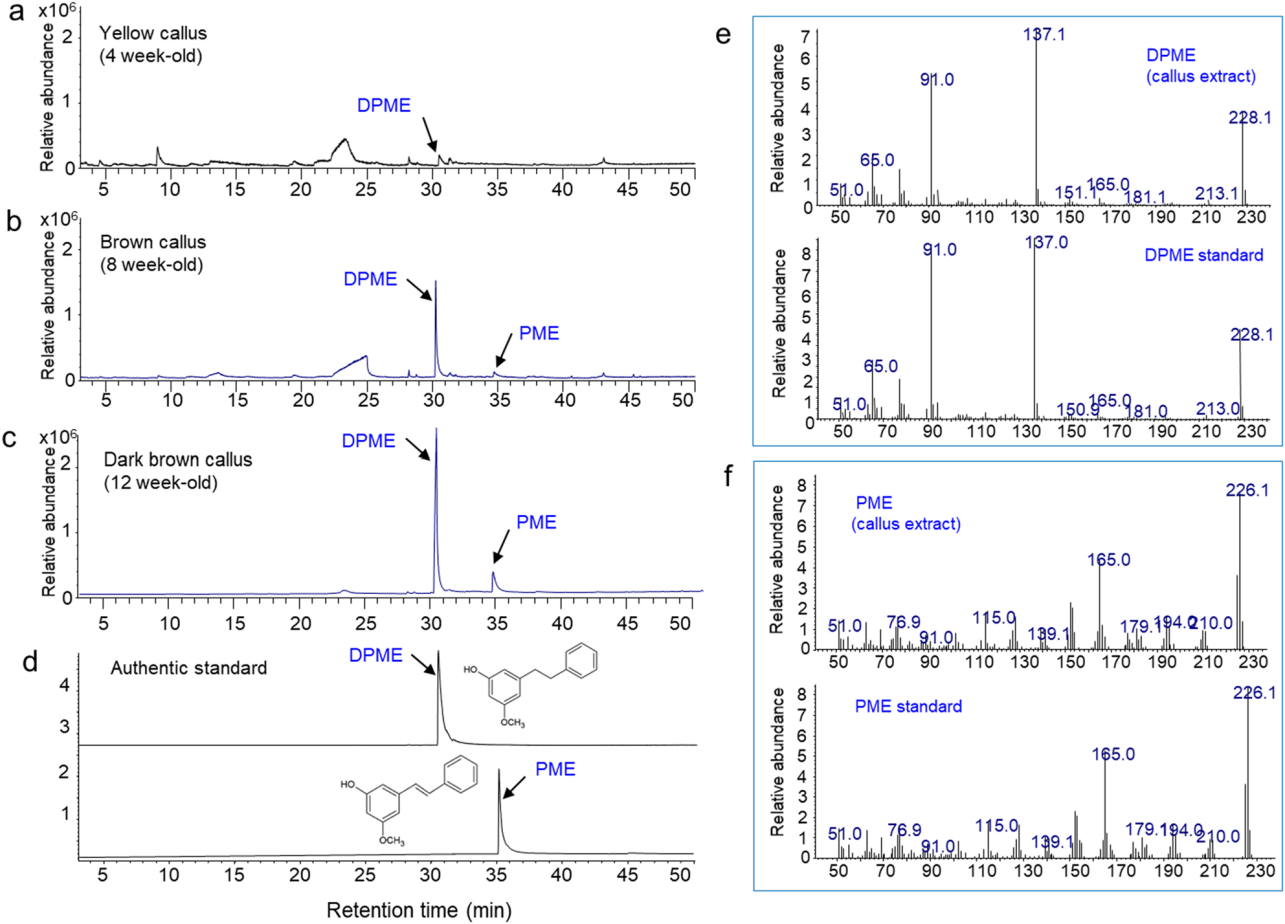
GC/MS analysis of the production of DPME and PME in *P. strobus* calli by prolonged culture. (**a**) GC chromatogram of extract from yellowish calli after 4 weeks of culture. (**b**) GC chromatogram of callus extract from pale brown callus after 8 weeks of culture. (**c**) GC chromatogram of callus extract from dark brown calli after 12 weeks of culture. (**d**) GC chromatogram of authentic standards of DPME and PME. (**e**) Mass fraction of a DPME peak in callus and DPME standard. (**f**) Mass fraction of a PME peak in callus and PME standard.

**Figure 6:**
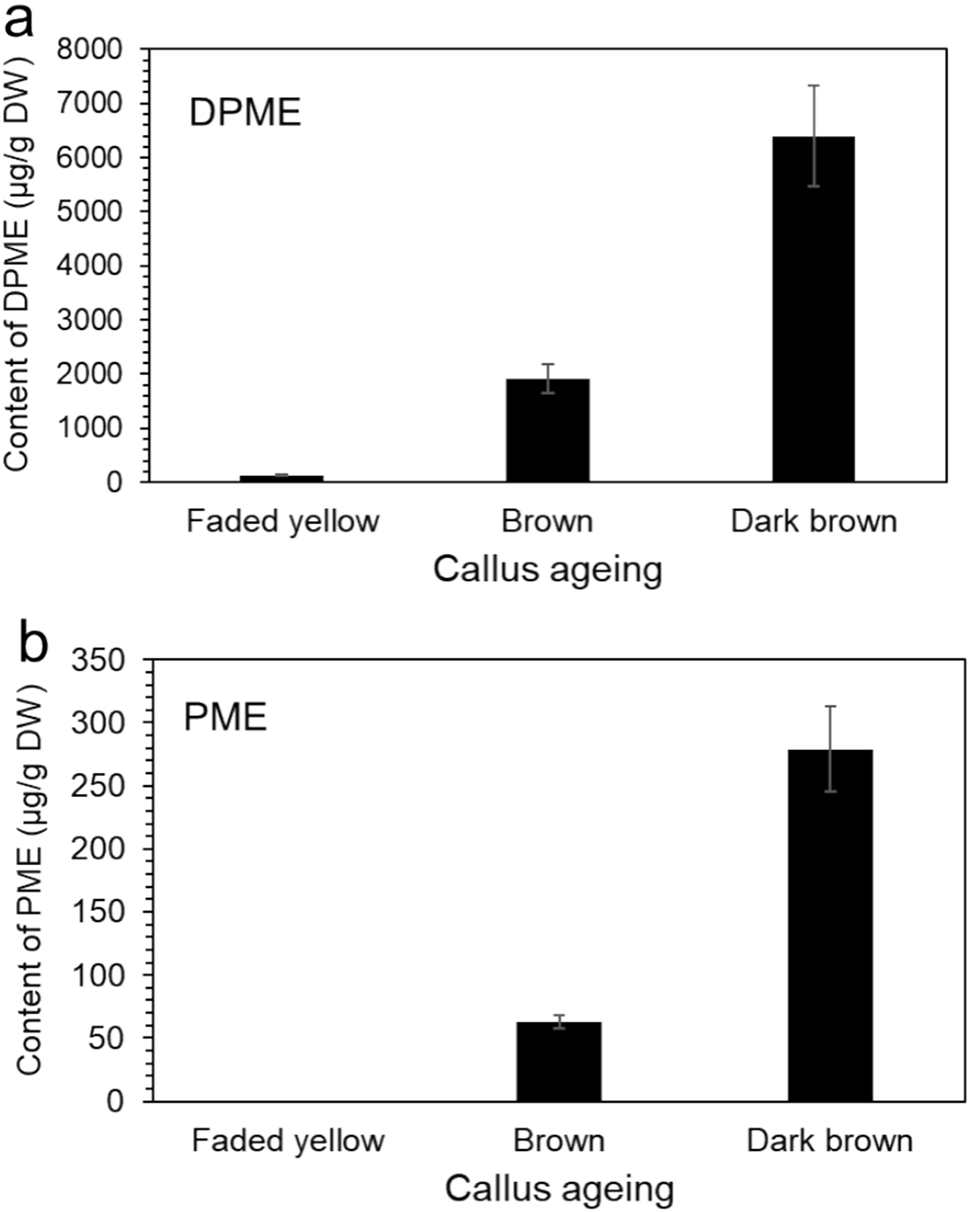
Enhanced accumulation of DPME and PME in *P. strobus* calli by prolonged incubation. The analysis results are presented as the means ± SEs of three independent experiments, each performed in technical triplicates.

Pinosylvin stilbene content in the MeOH extract of calli with different colors (faded yellow, brown, and dark brown) was analyzed by GC/MS. Interestingly, the accumulation of DPME and PME was highly increased by callus aging (Fig. 5). Only DPME was detected in a small amount, but PME was not at a detectable level in one-month-old calli with a faded yellow color (Fig. 5a). DPME and PME accumulations in calli were highly enhanced as the culture time proceeded to two and three months (Fig. 5b,c). The production of DPME and PME was confirmed by the retention times of authentic standards (Fig. 5d) and mass fraction pattern of the two compounds by GC/MS (Fig. 5e,f). Surprisingly, the total ion chromatogram over the full range of retention times revealed that the two DPME and PME peaks were the only major components in the extracts from dark brown calli (Fig. 5c). The amounts of DPME and PME in three-month-old calli with dark brown colors were 6.4 mg/g DW and 0.28 mg/g DW, respectively (Fig. 6).

### Effect of MeJA treatment of callus on DPME accumulation

**Figure 7:**
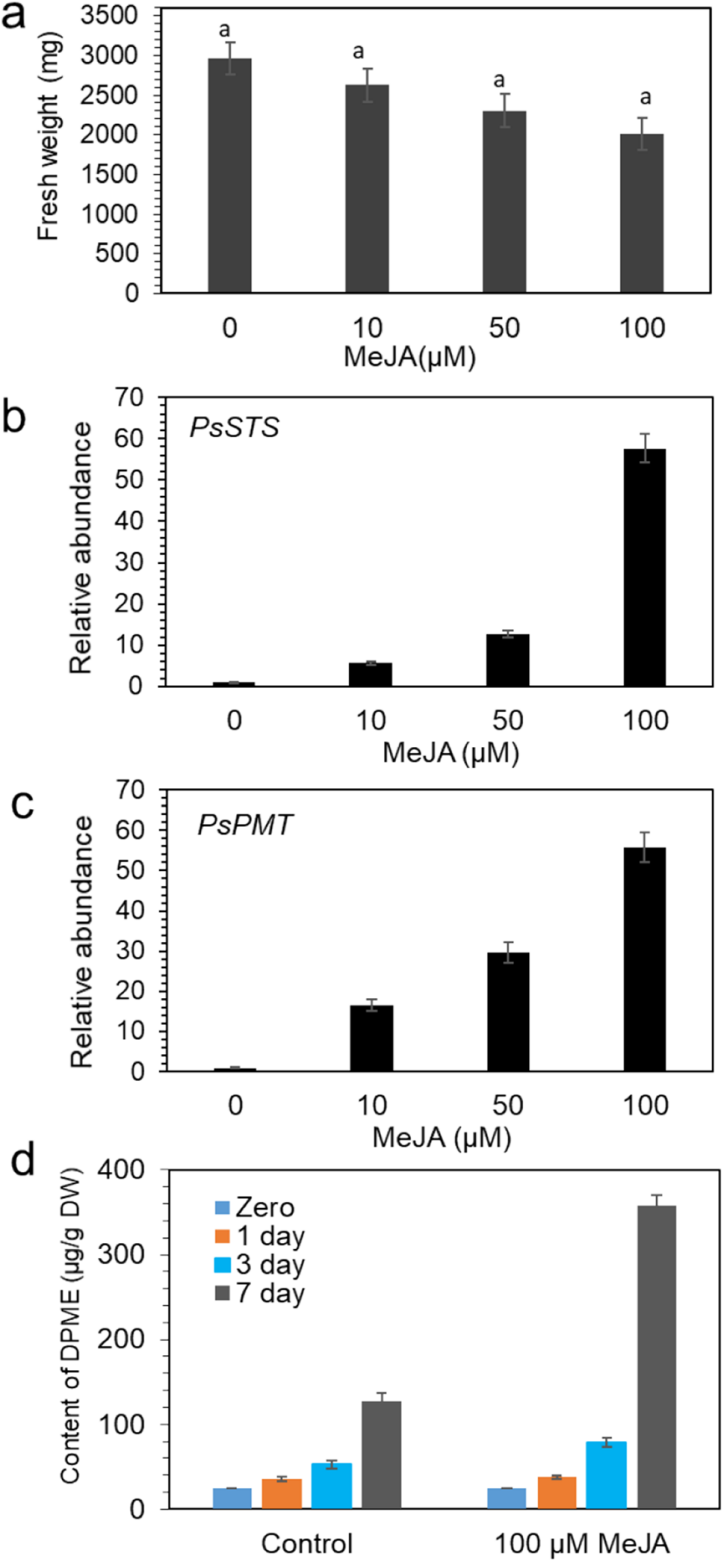
Growth of callus, accumulation of pinosylvin strilbene and expression of genes involved in pinosylvin strilbene biosynthesis in the different concentrations of MeJA. (**a**) Growth of calli grown in different concentrations of MeJA after 4 weeks of culture. (**b,c**) qPCR analysis of *PsSTS* and *PsPMT* genes in *P. strobus* callus. The qPCR data were normalized to β-actin gene expression. The vertical bars indicate the SE based on three biological replicates. (**d**) DPME accumulation in cell suspension during different days of culture in medium with or without 100 µM MeJA.

When *P. strobus* calli were transferred onto medium with MeJA at various concentrations (0, 10, 50, and 100 µM), callus growth after 2 weeks of culture was slightly suppressed by MeJA treatment, although there was no statistically significant difference (Fig. 7a). Pinosylvin synthase (STS) and pinosylvin O-methyltransferase (PMT) are key enzymes for pinosylvin stilbene biosynthesis in pine trees^13,37,38^. Previously, we selected the best candidate genes of the STS and PMT genes involved in pinosylvin stilbene biosynthesis in *P. strobus*^19^. The effect of MeJA treatment on the expression levels of *PsSTS* and *PsPMT* genes in calli was analyzed by qPCR. The expression of *PsSTS* and *PsPMT* was weak without MeJA treatment but significantly enhanced by MeJA treatment (Fig. 7b,c). Among the different concentrations (zero, 10, 50, and 100 µM MeJA treatment), the highest accumulation of *PsSTS* and *PsPMT* mRNAs was detected in calli after 100 µM MeJA treatment (Fig. 7b,c). The expression of *PsSTS* gene was more strongly responded by MeJA treatment than the *PsPMT.*

Cell suspension culture was obtained by shake flask culture of *P. strobus* calli in 1/2 LV liquid medium with 1.0 mg/L 2,4-D with 0.5 mg/L BA and with and without 100 µM MeJA. The accumulation of DPME and PME was monitored in the cell suspension culture during zero, 1, 3, and 7 days of culture. Without MeJA treatment, DPME accumulation was slightly increased as the culture period proceeded until 7 days (Fig. 7d, Supplemental Fig. S1a). Although the accumulation of DPME was more conspicuous in 100 µM MeJA treatment compared to control (Fig. 7d, Supplemental Fig. S1b), the amount of DPME (0.358 mg/g DW) in suspension cultured cells by 100 µM MeJA treatment for 7 days was 17.9 times lower than that of dark brown callus. Moreover, PME was under the detectable levels in suspensions cultured cells even regardless MeJA treatment (Supplemental Fig. S1b).

### Nematicidal activity of crude extracts from *P. strobus* calli

**Figure 8:**
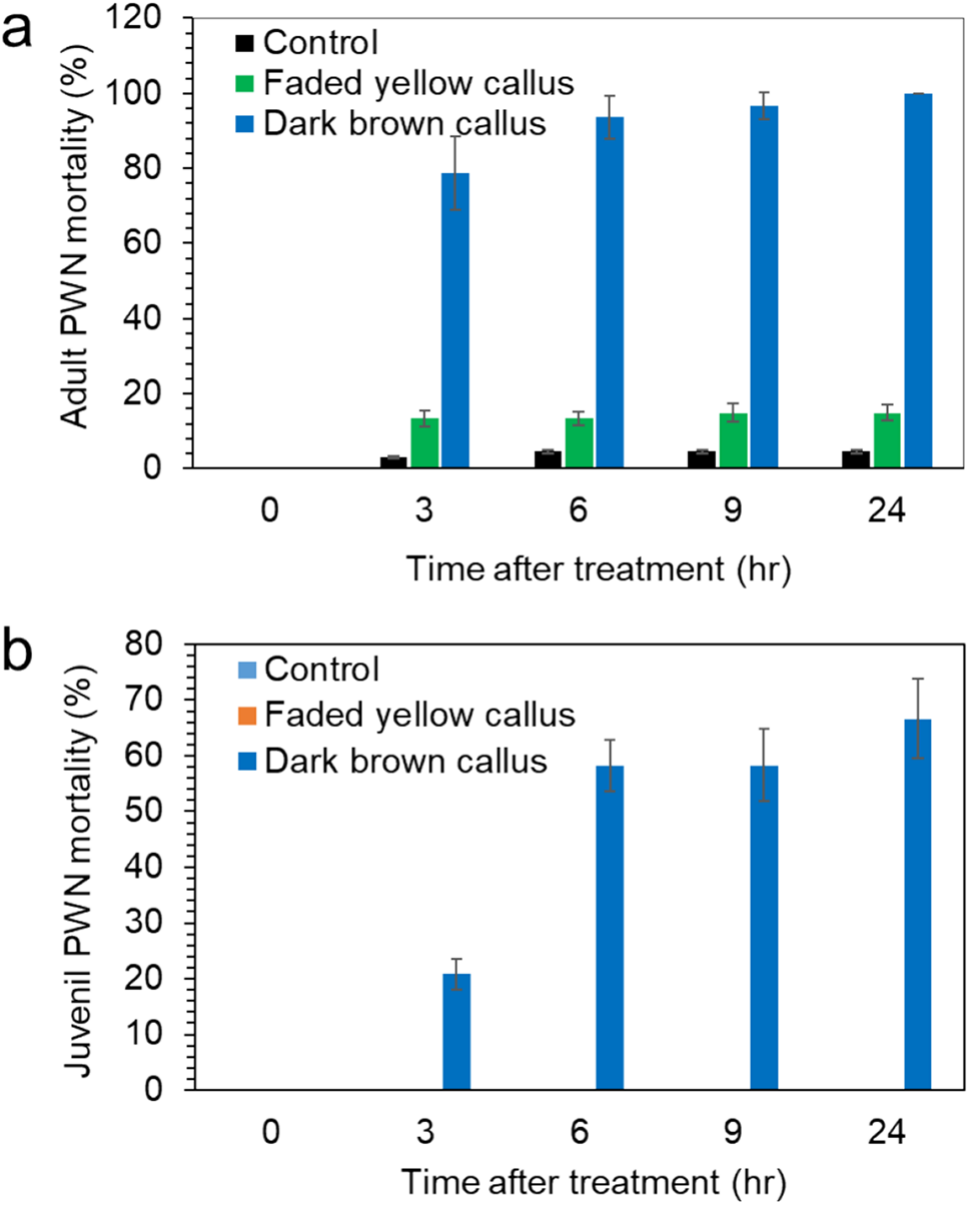
High nematicidal effects of *P. strobus* of aged dark brown callus extracts against PWNs. (**a**) Nematicidal effects of *P. strobus* callus extracts against adult PWNs during 24 h incubation. (**b**) Nematicidal effects of *P. strobus* callus extracts against juvenile PWNs during 24 h incubation. The concentrations of DPME and PME in the dark brown callus extract were adjusted to 120 µg/mL and 5.16 µg/mL, respectively.

Crude extracts were obtained by 100% EtOH extraction from calli of different ages (faded yellow and dark brown). The crude extracts were dissolved in water containing 10 mg/L 2-hydroxypropyl-β-cyclodextrin (HP-β-CD), which was used as an emulsifier of hydrophobic chemicals^39^. We previously demonstrated that the water soluble formulation of the pinosylvin stilbene:HP-β-CD complex was effective for the analysis of the nematocidal activity of DPME and PME compounds. The aqueous test solution of DPME and PME from extracts of dark brown callus was adjusted to the concentration of 120 µg/mL DPME (5.16 µg/mL PME) by dissolving in water containing HP-β-CD, and the same dilution was also applied for the crude extracts from faded yellow callus. The content of DPME and PME in faded yellow calli was nearly zero. These crude extracts were used to treat adult and juvenile PWNs. The extracts from dark brown calli showed strong nematicidal activity. More than 70% of adult PWNs lost their mobility with strait bodies after 3 h of callus extract treatment, and nearly all adult PWNs were immobilized after 24 h (Fig. 8a). However, the treated adult PWNs using the crude extracts from yellow calli did not exceed the immobilization of PWNs by less than 20% (Fig. 8a). In the control treatment (only water with HP-β-CD), less than 5% of PWNs showed immobilization of adult PWNs (Fig. 8a). When the crude extracts were treated with juvenile PWNs, the control water treatment and the extracts from faded yellow calli did not induce the immobilization of PWNs (Fig. 8b); only treatment with extracts from dark brown calli was effective for immobilization of PWNs at 67% after 24 h (Fig. 8b).

**Figure 9:**
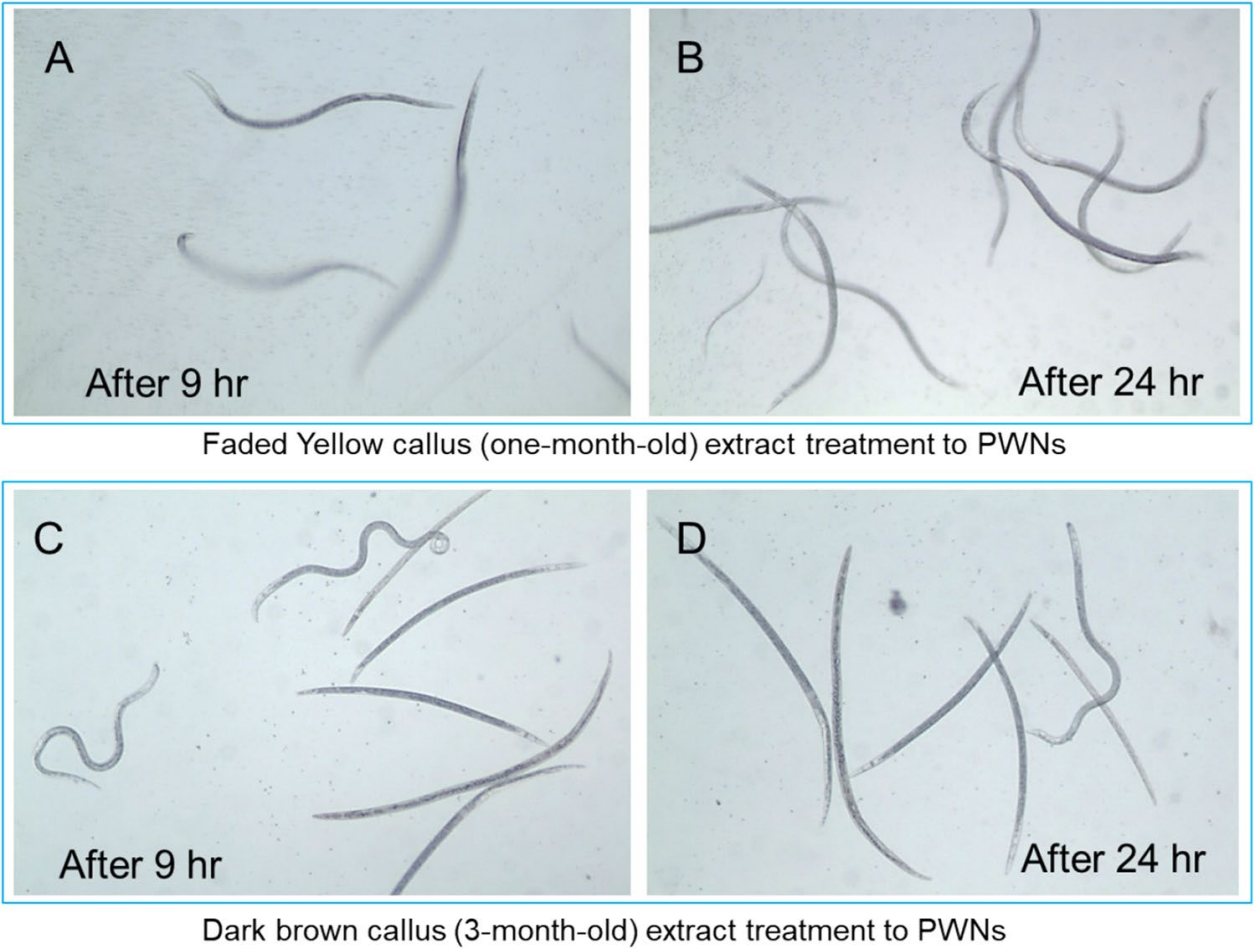
Photographs of PWN mobility after treatment with *P. strobus* callus extracts. (**a,b**) PWNs with active mobility after 9 h (**a**) and 24 h (**b**) of yellow callus extract treatment. (**c,d**) PWNs mixed with immobilized strait dead body shape after 9 h (**c**) and 24 h (**d**) of dark brown callus extract treatment.

Photos of PWNs revealed that all the PWNs showed active pendulation of their bodies by the extract treatment from yellow calli both after 9 h (Fig. 9a) and 24 h (Fig. 9b). In contrast, many PWNs rapidly lost their mobility at 9 h (Fig. 9c) and became strait body shaped due to immobilization at 24 h (Fig. 9d).

## Discussion

### Induction and proliferation of *P. strobus* callus

Pinosylvin stilbenes in the seedlings, bark, and sapwood of pine plants are not present or were present at low amounts and are only rich in knots^39^ and heartwood^9^. In three-year-old *P. strobus* plants, DPME and PME were either not or faintly detected under natural conditions but were highly enhanced after PWN infection^19^. Plant cell culture systems are an alternative method for producing secondary metabolites with industrial importance^20^. There is no report on the production of pinosylvin stilbenes in callus or cell suspension cultures of *P. strobus* except for a report on the production of pinosylvin stilbenes using *P. sylvestris* cell suspension cultures^26^. In this work, we attempt to investigate the culture conditions for the proliferation of calli and the production of pinosylvin stilbenes in *P. strobus* calli. We used LV medium to induce callus formation from mature zygotic embryos of *P. strobus*, which is one of the commonly used media for the cell culture of pine species^31^. The concentration of 2,4-D was effective for callus growth between 0.5 and 1 mg/L. Callus growth was stimulated by the addition of cytokinin (0.5 mg/L BA) to medium with 2,4-D (1.0 mg/L). There was no clear difference in the proliferation of calli among samples treated with different types of cytokinins. Generally, 2,4-D and BA are the most common plant growth regulators used for embryogenic callus induction and proliferation^40^. We found that the proliferation of nonembryogenic *P. strobus* calli actively occurred on medium with the combination of 2,4-D and BA, which is similar to the embryogenic callus culture reported in other pine species^40^.

### Enhanced DPME and PME accumulation by prolonged callus culture

There was no browning of the *P. strobus* callus in consecutive subcultures at 3-week intervals. However, the browning of callus color rapidly proceeded with prolonged culture without subculture. The fade yellow color of the callus turned dark brown after 3 months of culture. Generally, browning of calli is a common problem in plant tissue culture systems caused by the accumulation and oxidation of phenolic compounds^31,33^. We found that DPME and PME were not or faintly detected in nonbrown calli. Browning of the callus color commenced with prolonged culture. The accumulation of DPME and PME was highly enhanced in dark brown calli. The amounts of DPME and PME in dark brown calli at three months of age were 6.4 mg/g DW and 0.28 mg/g DW, respectively. These results indicate that callus aging strongly stimulates the production of pinosylvin stilbenes. The accumulation of phenolic pinosylvin stilbenes might be related to brown pigment accumulation. The accumulation of pinosylvin stilbenes accompanied with callus browning may be induced by cell death processes and induced by starvation of nutrients in the culture medium. In pine plants, normal undamaged sapwood does not contain pinosylvin stilbenes, which were found to be restricted to heartwood tissue without any living tree cells^41^. The accumulation of pinosylvin stilbenes in aged calli of *P. strobus* may be enhanced during cell death processes similar to the accumulation of pinosylvin stilbenes in heartwood tissues.

### Effect of MeJA on DPME and PME accumulation in *P. strobus* calli

Elicitor treatment using MeJA is effective for enhancing the production of resveratol (trans-3,5,4'-trihydroxystilbene) in grapevine cell culture^22–26^. We investigated the effect of MeJA on the accumulation of DPME and PME in a cell suspension culture of *P. strobus* and found that MeJA treatment only slightly stimulated DPME accumulation as the duration of culture proceeded until 7 days compared to the control. PME was undetectable inspite of MeJA treatment. This result indicates that the amount of DPME accumulation by MeJA treatment is much less (17.9 times lower) than by callus browning. Moreover, PME was not accumulated in suspensions cultured cells regardless MeJA treatment. Thus, callus browning is highly effective strategy for production of pinosylvin stilbenes in *P. strobus.* One report indicated that the production of pinosylvin stilbenes using *P. sylvestris* cell suspension cultures in which an elicitor treatment (prepared from the pine needle pathogen *Lophodermium seditiosum*) resulted in enhanced accumulation of pinosylvin and pinosylvin methyl ether^26^. Thus, fungal elicitors may be another effective treatment to enhance pinosylvin stilbene production by *P. strobus* calli and cell suspension culture.

### Strong nematicidal activity of diluted extracts from dark brown *P. strobus* callus

Recently, we reported that DPME and PME accumulation in *P. strobus* plants was highly enhanced by PWN infection, and the nematicidal activity of DPME and PME was different in adult and juvenile PWNs^19^. We prepared an aqueous test solution using ethanolic crude extracts from faded yellow and dark brown calli and investigated the nematicidal activity. Suga et al.^18^ reported that PME and DPME have strong nematicidal activity with 100% immobilization after 24 h of PWNs at concentrations of 10 µg/ml PME and 100 µg/ml DPME, respectively. We prepared an aqueous test solution at concentrations of 120 µg/mL DPME and 5.16 µg/mL PME using dark brown callus extracts. When the test solution containing DPME and PME using extracts from dark brown callus was treated to the PWNs, all the adult (100%) and juvenile (67%) PWNs were dead after 24 h. In contrast, most of the PWNs were alive by the treatment of the test solution using extracts from faded yellow callus. This result suggests that the aqueous test solution using dark brown callus extract has strong nematicidal activity, which might be related to the presence of DPME and PME.

In conclusion, we developed a protocol for establishing a callus culture system of *P. strobus*, and the production of pinosylvin stilbene in calli that effectively achieved by prolonged callus culture. The crude extracts of *P. strobus* calli showed strong nematicidal activity against PWNs. Thus, crude extracts from aged *P. strobus* calli can be used as ecofriendly agents with nematicidal activity against PWD. We will proceed with further experiments on the possible nematicidal activity of callus-derived pinosylvin stilbenes on PWN-susceptible pine tree plants.

## Supplementary Information

Supplementary Figure S1.

Supplementary Table S1.

## Data Availability

The original data presented in the study are included in the article/supplementary material, and further inquiries can be directed to the corresponding author.
