# Supplementary material for: Enhanced production of pinosylvin stilbene with aging of Pinus strobus callus and nematicidal activity of callus extracts against pinewood nematodes
Source: Sci Rep. 2022 Jan 14;12:770. doi: 10.1038/s41598-022-04843-6 (PMC8760238; doi:10.1038/s41598-022-04843-6)
Supplement: Supplementary file 1 — Supplementary Figure S1. [file 41598_2022_4843_MOESM1_ESM.pdf]

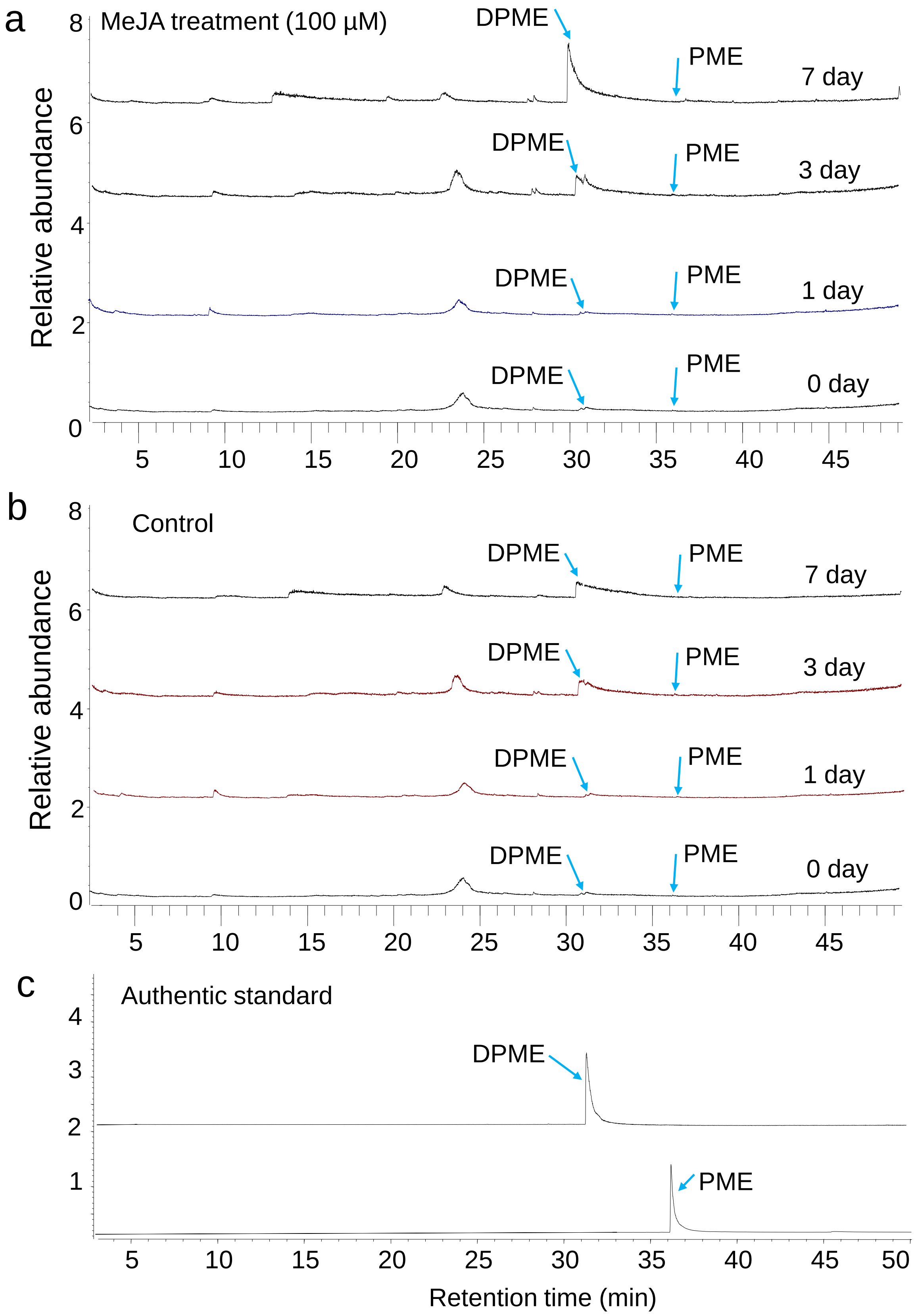

Supplemental Figure S1. GC chromatogram of cell extracts after zero, one, three, and seven days of cell suspension culture treated with (**A**) or without 100 mg/L MeJA (**B**). (**C**) GC chromatogram of standard DPME and PME compounds.
