# Supplementary material for: Enhanced production of pinosylvin stilbene with aging of Pinus strobus callus and nematicidal activity of callus extracts against pinewood nematodes
Source: Sci Rep. 2022 Jan 14;12:770. doi: 10.1038/s41598-022-04843-6 (PMC8760238; doi:10.1038/s41598-022-04843-6)
Supplement: Supplementary file 2 — Supplementary Table S1. [file 41598_2022_4843_MOESM2_ESM.pdf]

Supplemental Table S1. List of primers for qRT-PCR analysis involved in stilbene.

Gene	Primer direction	Sequence	Size (bp)
PsPMT (qPCR)	Forward	5'-GCC CTT TCC CGC ATT CTT TC-3'	163
	Reverse	5'-TGC TGA GAT TGG TAA GCC CG-3'	
β-actin (RT-PCR)	Forward	5'-CTT GCT GGG CGA GAT TTG AC-3'	102
	Reverse	5'-AGC TGT CTC AAG CTC CTG TTC-3'	
β-actin (qPCR)	Forward	5'-CTT GCT GGG CGA GAT TTG AC-3'	153
	Reverse	5'-AGC TGT CTC AAG CTC CTG TTC-3'	

biosynthesis.
